# Irregularities in the Number of Teeth

**Published:** 1858-09

**Authors:** W. H. Shadoan


### IRREGULARITIES IN THE NUMBER OF THE TEETH.

BY W. H. SHADOAN

MESSRS. EDITORS :—Having been somewhat familiar with the teeth, and being an operator on those particular organs, for a considerable time, I desire to note a few instances that have come immediately under my observation, thinking perhaps they will be of some interest to your readers and the profession generally. If you see anything new in the notes that I have taken, you will give them a place in your columns.

In last May, while on a visit to the town of Springville, Ind., there was a young man of some twenty-four or five years of age, called on me to have two incisors replaced, he having lost them some months previous—and also to have some filling done, which I did. On examining his teeth, I found that he had only one incisor remaining. I asked him if he did not want three teeth instead of two, this being the number gone. He said he did not, as he had lost but two teeth, therefore he only wanted the two. I told him he certainly was mistaken, I had never seen a full denture unless there were four incisors, and I thought that as nature had given him his complement of grinding teeth, that she certainly had not failed to give him his share of cutting teeth. He contended that he was right, and said he knew better than I did. I then examined his inferior set, and found them as he said his superior set was, only three incisors. There happened to be a practicing physician in at the time, I called his attention to the teeth. He remarked he had never seen such a deficiency in the teeth, and had been practicing medicine for a number of years and had never seen such a case before. The position of the teeth was, the central incisor (not incisors) was in the center, and the laterals on each side.

2nd. I was called on a few days ago to extract the cuspids for a young man of eighteen or nineteen years of age, they having been crowded out by the incisors. I examined the teeth and found that he had six incisors all well set; and very regular in line. I extracted the cuspids and left him with his full number of teeth, and he went off well pleased. I was of opinion at first that he still retained two of his temporary teeth, but on inquiring, I found that he had lost them some years previous.

The question arises, were there four lateral pulps contained in the two sacs, or were there four sacs containing the germs for these four lateral incisors, while he only had his complement of temporary teeth, or did two teeth come from one pulp? I would like to know. I have thought of this some, and have come to the conclusion that the sacs containing the pulps for the cuspids had each two pulps, one incisor and one cuspid, as the incisor that occupied the place that the cuspidata should, one being straight, and to all appearances was intended for that place.

ROCKPORT, IND.

---

WE have occasionally seen variations in the number of the teeth, more frequently in the absence of wisdom teeth than any other class, the next class most frequently affected thus is the incisors. In quite a number of instances we have found but three inferior incisors; and in some cases but three, and even two superior incisors. In two three or cases, we have seen a hereditary deficiency, running entirely through a family. The cause may originate in two or three ways, the germ in some cases is never formed, then the position of the teeth will be regular, all having a perfect position. But if a germ once existed, and was destroyed by any injurious influence, the teeth will be irregular to some extent. There are various influences that may destroy the germs of the permanent teeth. Constitutional disturbances, particularly those of an eruptive character, may accomplish this. Why the germs of the teeth are sometimes not produced, we can not tell, except, as in many other instances, nature fails to perform the work. ED.

